# Real-Time Implementation of the Prescribed Performance Tracking Control for Magnetic Levitation Systems

**DOI:** 10.3390/s22239132

**Published:** 2022-11-24

**Authors:** Thanh Nguyen Truong, Anh Tuan Vo, Hee-Jun Kang

**Affiliations:** Department of Electrical, Electronic and Computer Engineering, University of Ulsan, Ulsan 44610, Republic of Korea

**Keywords:** prescribed performance tracking control, terminal sliding mode control, disturbance observer, magnetic levitation systems

## Abstract

For magnetic levitation systems subject to dynamical uncertainty and exterior perturbations, we implement a real-time Prescribed Performance Control (PPC). A modified function of Global Fast Terminal Sliding Mode Manifold (GFTSMM) based on the transformed error of the novel PPC is introduced; hence, the error variable quickly converges to the equilibrium point with the prescribed performance, which means that maximum overshoot and steady-state of the controlled errors will be in a knowledge-defined boundary. To enhance the performance of Global Fast Terminal Sliding Mode Control (GFTSMC) and to reduce chattering in the control input, a modified third-order sliding mode observer (MTOSMO) is proposed to estimate the whole uncertainty and external disturbance. The combination of the GFTSMC, PPC, and MTOSMO generates a novel solution ensuring a finite-time stable position of the controlled ball and the possibility of performing different orbit tracking missions with an impressive performance in terms of tracking accuracy, fast convergence, stabilization, and chattering reduction. It also possesses a simple design that is suitable for real-time applications. By using the Lyapunov-based method, the stable evidence of the developed method is fully verified. We implement a simulation and an experiment on the laboratory magnetic levitation model to demonstrate the improved performance of the developed control system.

## 1. Introduction

The potential applications of magnetic levitation systems are huge. They have become quite popular as a testing system in control engineering labs and advanced nonlinear control programs. A major reason for the popularity of this nonlinear system is the fact that it is relatively easy to construct and manage. Their many applications can be found in real systems such as contactless melting, rocket-guiding projects, gyroscopes, high-speed trains, frictionless bearings, vibration isolation systems, etc. The characteristics of Magnetic Levitation Systems (MLSs) tend towards nonlinearity and instability described by nonlinear differential formulas. The proportional-integral-differential (PID) and proportional-derivative (PD) controllers are typically proposed for the effective regulation of systems under the assumption of well-known gain parameters [1]. The tracking control performance of those controllers can rapidly deteriorate as deviations from their nominal operating point increase. There is no doubt that the nonlinearity and inherent instabilities of the system prevent classical PD or PID controllers from being applied to these more complex problems. A trajectory tracking task typically involves the gain of the system no longer being constant, and it is determined by changes in distance from the magnet. In order to maintain operating time with a high steady-state as long as possible, advanced control methods, such as nonlinear controllers [2,3,4,5,6], should be applied to MLSs. Ref. [7] developed a nonlinear controller based on fast online algebraic identification of the input gain for an MLS. Unfortunately, this controller was not considered the effects of external disturbances, and it only guarantees an asymptotically exponentially stable. In addition, variation factors, such as system parameters, resistance, inductance, and suspending mass, should be taken into account. The implemented algorithms in real-time systems about the issue of tracking output trajectory tasks for the suspended metal ball did not use trajectories of certain difficulty to produce controlled motions such as sinusoidal or rest-to-rest reference trajectory positions. Thus, the verification is not general. Most of the methods introduced for MLS achieve only asymptotic stability, and a few achieve finite-time stability. However, achieving prescribed performance has hardly been introduced for MLSs in the literature. Therefore, there is still much interest in improving the performance of MLSs.

In order to handle the influences of uncertainties and exterior disturbances, sliding mode control (SMC) with powerful and immutable properties can be employed effectively. In recent years, SMC has been successfully applied for a wide range of practical applications such as unmanned aerial vehicles (UAVs) [8,9,10], autonomous underwater vehicles (AUVs) [11,12,13,14,15], robotic manipulators [16,17,18,19,20], and so on. In the approach stage, SMC cannot maintain uniform characteristics due to the existence of unidentified uncertain elements. Moreover, the SMC’s control performance depends on the linear sliding mode surface and a discontinuous control law to drive the state variables to that sliding surface. Consequently, this method only has an asymptotically exponentially stable along with a non-smooth control signal that is known as chattering. Whenever higher accuracy and faster convergence are needed under the SMC method, a very large control force is required, which is not possible because the capability of the hardware devices is limited, and chattering behavior also becomes much more serious.

Terminal SMC (TSMC) [21], Fast TSMC [22], GFTSMC [16,23], Non-singular TSMC [24], or Non-singular Fast TSMC (NFTSMC) [25] were proposed to inherit the invariant properties of the SMC and at the same time overcome the SMC’s disadvantages. Several attractive properties of these methods include robustness against uncertain terms, finite-time convergence, and high accuracy. Thus far, these methods have also been successfully applied to real-time nonlinear systems in general and MLSs in particular. However, each method also has its disadvantages. For example, slow convergence speed, singularity, and chattering are the main weaknesses of TSMC, while singularity and chattering are the main weaknesses of Fast TSMC. The main weakness of the two remaining control methods is chattering. The issue of chattering attenuation has therefore become a popular one. Moreover, according to the knowledge of the authors, there are currently no studies related to the prescribed performance control based on the aforementioned methods proposed for MLSs. This is also one of the main motivations of this article.

The use of quasi-sliding mode control, neural-network-based SMC methods [6], disturbance observers [26,27], super-twisting SMC methods [28,29], second-order SM observers (SOSMOs) [30], third-order SM observers (TOSMOs) [17,31], and so on have been proposed for resolving the chattering. It is noted that the active control methods have been proven to provide better tracking performance if the uncertain components are approximated correctly when compared to the passive control methods. For example, the technique of applying an observer to reduce chattering behavior can be understood as a disturbance observer will approximate the uncertain components to obtain a precise dynamic model. As a consequence, the approximate error from the observer can only be compensated by using a reasonable sliding gain. Among the observers discussed, TOSMO is superior to the rest when convergence is achieved in finite time and only needs information from the position sensor. However, its convergence speed also needs to be further improved to avoid causing delays in the control system.

In our paper, an MLS suspends a metal ball in the air by electromagnetic force. The ball and MLS do not interact mechanically. It is inherently unstable and highly nonlinear. Adjusting and tracking the reference trajectory of the levitated ball is a highly challenging process. Therefore, the control target is to develop a new real-time prescribed performance tracking controller for MLSs under the effects of uncertainty and exterior disturbance. The important contributions of the proposed methods can be listed below:

- A modified TOSMO is applied to quickly estimate the approximate value of the uncertainty and exterior disturbance;
- The novel Prescribed Performance Function (PPF) does not contain a singularity problem and can flexibly adjust lower and upper bounds. Furthermore, it can extend the operation domain at a steady state compared to that of the conventional PPF. With the proposed PPF, the steady-state error boundaries will be symmetric to zero, so, when the transformed error converges to zero, the tracking error also converges to zero;
- A modified function of GFTSMM based on the transformed errors of the PPC is introduced; hence, the error variables quickly converge to the equilibrium point with the prescribed performance;
- The maximum overshoot, convergence index, and steady-state error can be managed within a predefined domain under the proposed controller;
- A novel solution ensures a finite-time stable position of the controlled ball and the possibility of performing different orbit tracking missions with an impressive performance in the terms of tracking accuracy, fast convergence, stabilization, and chattering reduction;
- The effectiveness of the designed control solution was confirmed by simulation and experiment;
- This controller is presented in a way that can be applied to real-time applications. In addition, it can apply not only to MLSs but also to a class of second-order nonlinear systems.

Our manuscript is structured as follows: The formulation of the problem takes place in Section 2. The control method design is discussed in Section 3. Section 4 is the simulation and experimental results. Section 5 is conclusions.

## 2. Problem Statements

**Figure 1:**
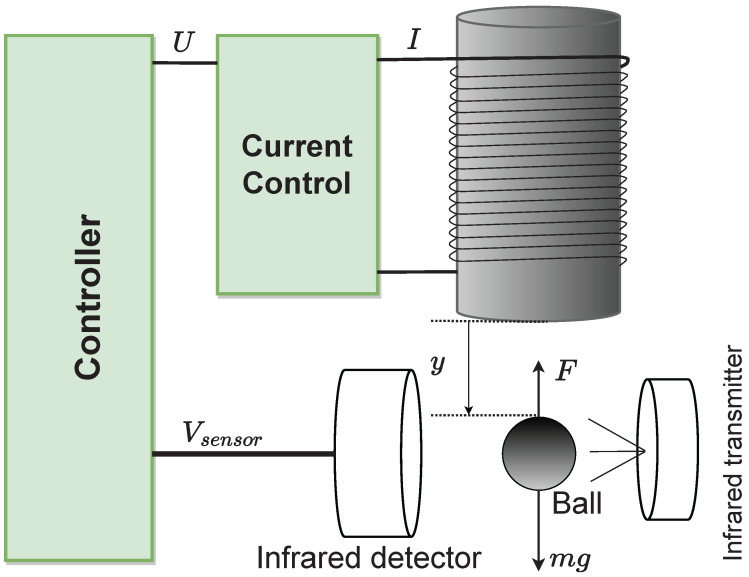
Magnetic levitation system graph.

The structure of the MLS is depicted in Figure 1. In addition, an explanation of this MLS is provided in the article [4].

The dynamic model of the MLS is described as:

(1) $$m\ddot{y}=mg-\lambda {\left(\frac{I}{y}\right)}^{2}$$

(2) I=KU

where *m* is the mass of the levitated ball, *g* denotes the acceleration due to gravity, *U* is the control input voltage, *I* is the winding current, *y* is the position of the levitated ball, *K* is the constant related to the input voltage and the current through the coil, and λ is a constant related to the mutual inductance of the ball and coupling coefficients.

Substituting Equation (2) into Equation (1) yields:

(3) $$\ddot{y}=g-\frac{\mu U^{2}}{y^{2}}$$

where $\mu =\frac{\lambda K^{2}}{m}$.

The exact value of μ cannot be known, it can be identified by using an identification method such as [7]. In addition, considering the effects of uncertainty and exterior disturbance, Equation (3) can be described as:

(4) $$\ddot{y}=g-\frac{\hat{\mu }U^{2}}{y^{2}}+\Delta \left(y,\delta ,t\right),$$

where $\hat{\mu }$ represents the estimated value of μ, and $\Delta \left(y,\delta ,t\right)=\frac{\mu -\hat{\mu }}{y^{2}}U^{2}+\delta \left(t\right)$ is a function of exterior disturbance and interior uncertainty, δ(t) is exterior disturbance. Let $y_{1}=y$, and $y_{2}=\dot{y}$; then, Equation (4) is rewritten as:

(5) $$\left\{\begin{array}{c} {\dot{y}}_{1}=y_{2}\\ {\dot{y}}_{2}=g-\frac{\hat{\mu }U^{2}}{y_{1}^{2}}+\Delta \left(y,\delta ,t\right) \end{array}\right.$$

**Assumption** **1.** *It is assumed that uncertain terms are bound by the following:*

(6) $$\left|\Delta \left(y,\delta ,t\right)\right|\leq \bar{\Delta },$$

*where $\bar{\Delta }$ is a positive constant.*

MLS suspends a metal ball in the air by electromagnetic force. The ball and MLS do not interact mechanically. It is inherently unstable and highly nonlinear. Adjusting and tracking the reference trajectory of the levitated ball is a highly challenging process. Therefore, the objective of our paper is to develop a new real-time prescribed performance tracking controller for MLSs under the effects of uncertainty and exterior disturbance ensuring a finite-time stable position of the controlled ball and the possibility of performing different orbit tracking missions with an impressive performance in the terms of tracking accuracy, fast convergence, stabilization, and chattering reduction.

## 3. Design of the Proposed Control Method

### 3.1. Design of the Sliding Mode Surface

The position error and velocity error are respectively defined as $\tilde{y}=y_{1}-y_{r}$ and $\dot{\tilde{y}}={\dot{y}}_{1}-{\dot{y}}_{r}$, where $y_{r}$ and ${\dot{y}}_{r}$ represent the reference trajectory and its derivative.

For system (5), the sliding mode surface is constructed by using the position error and velocity error, as follows:

(7) $$s=\dot{\tilde{y}}+\lambda^{*}sig{\left(\tilde{y}\right)}^{\alpha }+\omega^{*}sig{\left(\tilde{y}\right)}^{\beta }$$

where $\lambda^{*}=\frac{2\lambda }{1+e^{-\eta \left(\left|\tilde{y}\right|-\gamma \right)}}$, $\omega^{*}=\frac{2\omega }{1+e^{\vartheta \left(\left|\tilde{y}\right|-\gamma \right)}}$, λ>0,η>0,ω>0,ϑ>0, α>1, 0<β<1, $\gamma ={\left(\omega /\lambda \right)}^{1/\left(\alpha -\beta \right)}$, and the $sig{\left(\right)}^{k}$ function is defined as $sig{\left(x\right)}^{k}={\left|x\right|}^{k}sign\left(x\right)$ with k>0.

The finite-time stable evidence of the selected sliding manifold is given in [23]. The convergence time $T_{s}$ of the sliding mode motion is stated in Equation (27) of [23].

### 3.2. Design of Global Fast Terminal Sliding Mode Control

Using the selected sliding mode surface, we can calculate its time derivative as follows:

(8) $$\begin{array}{c} \dot{s}=\ddot{\tilde{y}}+\frac{2\lambda \alpha }{1+e^{-\eta \left(\left|\tilde{y}\right|-\gamma \right)}}{\left|\tilde{y}\right|}^{\alpha -1}\dot{\tilde{y}}+\frac{2\lambda \eta e^{-\eta \left(\left|\tilde{y}\right|-\gamma \right)}}{{\left(1+e^{-\eta \left(\left|\tilde{y}\right|-\gamma \right)}\right)}^{2}}{\left|\tilde{y}\right|}^{\alpha }\dot{\tilde{y}}\\ \;\;\;\;\;\;\;+\frac{2\omega \beta }{1+e^{\vartheta \left(\left|\tilde{y}\right|-\gamma \right)}}{\left|\tilde{y}\right|}^{\beta -1}\dot{\tilde{y}}-\frac{2\omega \vartheta e^{\vartheta \left(\left|\tilde{y}\right|-\gamma \right)}}{{\left(1+e^{\vartheta \left(\left|\tilde{y}\right|-\gamma \right)}\right)}^{2}}{\left|\tilde{y}\right|}^{\beta }\dot{\tilde{y}} \end{array}$$

Equation (8) is rewritten as:

(9) $$\dot{s}=\ddot{\tilde{y}}+M_{\tilde{y}}={\dot{y}}_{2}-{\ddot{y}}_{r}+M_{\tilde{y}}$$

where $M_{\tilde{y}}=\frac{2\lambda \alpha }{1+e^{-\eta \left(\left|\tilde{y}\right|-\gamma \right)}}{\left|\tilde{y}\right|}^{\alpha -1}\dot{\tilde{y}}+\frac{2\lambda \eta e^{-\eta \left(\left|\tilde{y}\right|-\gamma \right)}}{{\left(1+e^{-\eta \left(\left|\tilde{y}\right|-\gamma \right)}\right)}^{2}}{\left|\tilde{y}\right|}^{\alpha }\dot{\tilde{y}}+\frac{2\omega \beta }{1+e^{\vartheta \left(\left|\tilde{y}\right|-\gamma \right)}}{\left|\tilde{y}\right|}^{\beta -1}\dot{\tilde{y}}-\frac{2\omega \vartheta e^{\vartheta \left(\left|\tilde{y}\right|-\gamma \right)}}{{\left(1+e^{\vartheta \left(\left|\tilde{y}\right|-\gamma \right)}\right)}^{2}}{\left|\tilde{y}\right|}^{\beta }\dot{\tilde{y}}$.

Substituting system (5) into Equation (9) yields:

(10) $$\dot{s}=g-\frac{\hat{\mu }}{y_{1}^{2}}U^{2}+\Delta \left(y,\delta ,t\right)-{\ddot{y}}_{r}+M_{\tilde{y}}$$

From Equation (10), GFTSMC is designed as:

(11) $$U=\sqrt{\frac{y_{1}^{2}}{\hat{\mu }}\left(u_{eq}+u_{r}\right)}$$

in which the terms of $u_{eq}$ and $u_{r}$ are designed as:

$$\begin{array}{c} \left\{\begin{array}{c} u_{eq}=g+M_{\tilde{y}}-{\ddot{y}}_{r}\\ u_{r}=\sigma_{1}s+\left(\bar{\Delta }+\kappa_{1}\right)\mathrm{sign}\left(s\right) \end{array}\right. \end{array}$$

where $\kappa_{1}$ and $\sigma_{1}$ are positive constants.

The following evidence will be given to prove the GFTSMC’s correctness and stability.

Evidence: Applying the control signal system (11) to Equation (10), we have:

(12) $$\dot{s}=\Delta \left(y,\delta ,t\right)-\left(\bar{\Delta }+\kappa_{1}\right)\mathrm{sign}\left(s\right)-\sigma_{1}s$$

Considering the Lyapunov function $V_{1}=0.5s^{2}$, then we see that:

(13) $${\dot{V}}_{1}=s\dot{s}$$

By substituting Equation (12) into Equation (13), we obtain:

(14) $$\begin{array}{c} {\dot{V}}_{1}=\Delta \left(y,\delta ,t\right)s-\bar{\Delta }\left|s\right|-\kappa_{1}\left|s\right|-\sigma_{1}s^{2}\\ \;\;\;\;\;\;\;\;\leq -\kappa_{1}\left|s\right|-\sigma_{1}s^{2}\leq 0 \end{array}$$

Obviously, ${\dot{V}}_{1}\leq 0$ and $V_{1}⩾0$. It means that the sliding variables $s,\dot{s}$ can reach the sliding manifold (s→0 and $\dot{s}\to 0$), and $\tilde{y},\dot{\tilde{y}}$ can reach the equilibrium point.

Generally, it is hard to provide an exact function to the nominal controller by offering a mathematical model of dynamical uncertainties and disturbances. In addition, the designed controller (11) does not provide a prescribed controlled performance. This means that the maximum overshoot, convergence index, and steady-state error are not managed within a predefined domain. To overcome the mentioned challenges, a modified observer is applied to quickly estimate the approximate value of the uncertainty and exterior disturbance while the proposed controller is designed based on PPC to achieve the desired-prescribed control performance.

### 3.3. Design of a Disturbance Observer for Magnetic Levitation Systems

In this section, a modified TOSMO is designed to estimate the whole interior uncertainty and exterior disturbance. This observer is designed based on the modification of TOSMO [32] to improve the convergence speed of TOSMO as follows:

(15) $$\left\{\begin{array}{c} {\dot{\hat{y}}}_{1}=\pi_{1}{\left[{\bar{y}}_{1}\right]}^{{}^{\frac{2}{3}}}+\rho {\bar{y}}_{1}+{\hat{y}}_{2}\\ {\dot{\hat{y}}}_{2}=g-\frac{\hat{\mu }}{y_{1}^{2}}U^{2}+\pi_{2}{\left[{\bar{y}}_{1}\right]}^{{}^{\frac{1}{3}}}\\ \;\;\;\;\;\;\;\;\;\;+\rho \left({\dot{\hat{y}}}_{1}-{\hat{y}}_{2}\right)+\hat{\Delta }\\ \dot{\hat{\Delta }}=\pi_{3}sign\left({\bar{y}}_{1}\right) \end{array}\right.$$

where ${\hat{y}}_{1}$, ${\hat{y}}_{2}$, and $\hat{\Delta }$ are the estimated value of $y_{1}$, $y_{2}$, and $\Delta \left(y,\delta ,t\right)$, respectively. $\pi_{i}\left(i=1,2,3\right)$ represents observer gain which is selected as [33], and ρ is a design positive constant. By selecting the suitable design parameter ρ, the convergence rate of the modified TOSMO (15) can be improved immensely.

We can obtain the estimation error by subtracting Equation (15) from Equation (5) as follows:

(16) $$\left\{\begin{array}{c} {\dot{\bar{y}}}_{1}=-\pi_{1}{\left[{\bar{y}}_{1}\right]}^{{}^{\frac{2}{3}}}-\rho {\bar{y}}_{1}+{\bar{y}}_{2}\\ {\dot{\bar{y}}}_{2}=-\pi_{2}{\left[{\bar{y}}_{1}\right]}^{{}^{\frac{1}{3}}}-\rho \pi_{1}{\left[{\bar{y}}_{1}\right]}^{\frac{2}{3}}-\rho^{2}{\bar{y}}_{1}\\ \;\;\;\;\;\;\;\;\;\;\;\;+\Delta \left(y,\delta ,t\right)-\hat{\Delta }\\ \dot{\hat{\Delta }}=\pi_{3}sign\left({\bar{y}}_{1}\right) \end{array}\right.$$

where ${\bar{y}}_{1}=y_{1}-{\hat{y}}_{1}$, ${\bar{y}}_{2}=y_{2}-{\hat{y}}_{2}$. Define $X={\bar{y}}_{2}-\rho {\bar{y}}_{1}$ and use Equation (16), therefore,

(17) $$\begin{array}{c} \dot{X}={\dot{\bar{y}}}_{2}-\rho {\dot{\bar{y}}}_{1}\\ \;\;\;\;\;=-\pi_{2}{\left[{\bar{y}}_{1}\right]}^{\frac{1}{3}}-\rho \pi_{1}{\left[{\bar{y}}_{1}\right]}^{\frac{2}{3}}-\rho^{2}{\bar{y}}_{1}\\ \;\;\;\;\;\;\;\;\;+\Delta \left(y,\delta ,t\right)-\hat{\Delta }-\rho \left(-\pi_{1}{\left[{\bar{y}}_{1}\right]}^{\frac{2}{3}}-\rho {\bar{y}}_{1}+{\bar{y}}_{2}\right)\\ \;\;\;\;\;=-\pi_{2}{\left[{\bar{y}}_{1}\right]}^{\frac{1}{3}}+\Delta \left(y,\delta ,t\right)-\hat{\Delta }-\rho {\bar{y}}_{2} \end{array}$$

Using Equation (17), Equation (16) can be rewritten in form of the TOSMO as follows:

(18) $$\left\{\begin{array}{c} {\dot{\bar{y}}}_{1}=-\pi_{1}{\left[{\bar{y}}_{1}\right]}^{\frac{2}{3}}+X\\ \dot{X}=-\pi_{2}{\left[{\bar{y}}_{1}\right]}^{\frac{1}{3}}+Z\\ \dot{Z}=-\pi_{3}sign\left({\bar{y}}_{1}\right)+\dot{\Sigma } \end{array}\right.$$

where $\Sigma =\Delta \left(y,\delta ,t\right)-\rho {\bar{y}}_{2}$ and $Z=\Sigma -\hat{\Delta }$. Suppose that $\left|\frac{d\Sigma }{dt}\right|\leq \bar{\Sigma }$.

Selecting the positive Lyapunov function and performing the proof process like [33], we concluded that the TOSMO (18) is stable and the differentiators including ${\bar{y}}_{1}$, *X*, and *Z* reach zero in finite time. Consequently, the proposed observer (15) is finite-time stable, and the estimate errors reach zero in finite time.

### 3.4. Prescribed Performance Control

**Remark** **1.** *Most of the existing studies of PPC [34,35,36,37,38,39] use only one prescribed performance function (PPF) to generate specified performance boundaries, for example [34], p(t) is a PPF, the upper boundary can be p(t), and the lower boundary can be −Lp(t)(0<L<1). We can clearly see that generating boundaries from a PPF will have disadvantages, such as the lower boundary will be L times smaller than the upper boundary at steady-state, which makes the operating area of specified performance be scaled down over a specified static error value (e.g., $p_{\infty }$ is the specified error value of tracking error at steady-state, the upper boundary value specified as $p_{\infty }$ and the lower boundary value specified as $-Lp_{\infty }$ at steady-state). As a result, the upper and lower boundaries will not be symmetrical about zero at the steady state, and this leads to the fact that, although the transformed error equals zero, the tracking error deviates from zero. Using a ratio of a PPF to create the lower boundary makes it more difficult to choose the error transformation function (ETF) (due to the unsymmetrical about zero of the boundaries at steady-state). In addition, some ETFs [34,40,41,42] have a singularity problem, which seriously affects the operation of the real system.*

In our paper, we propose two separate PPFs to assign each of the upper and lower bounds of the prespecified performance for tracking error, in which one PPF limits the convergence rate and steady-state error, and the other PPF limits the overshoot and steady-state error on the other side. Setting the same allowable range of tracking error at the steady state of two PPFs makes the specified performance space at the steady-state larger than in the traditional way. Moreover, the steady-state error boundaries will be symmetric to zero, so when the transformed error is zero, the tracking error is also zero. ETFs can be designed more easily using the above design. In addition, we design an ETF that does not suffer from singularity problems. Consequently, the stated drawbacks in Remark 1 have been overcome.

**Figure 2:**
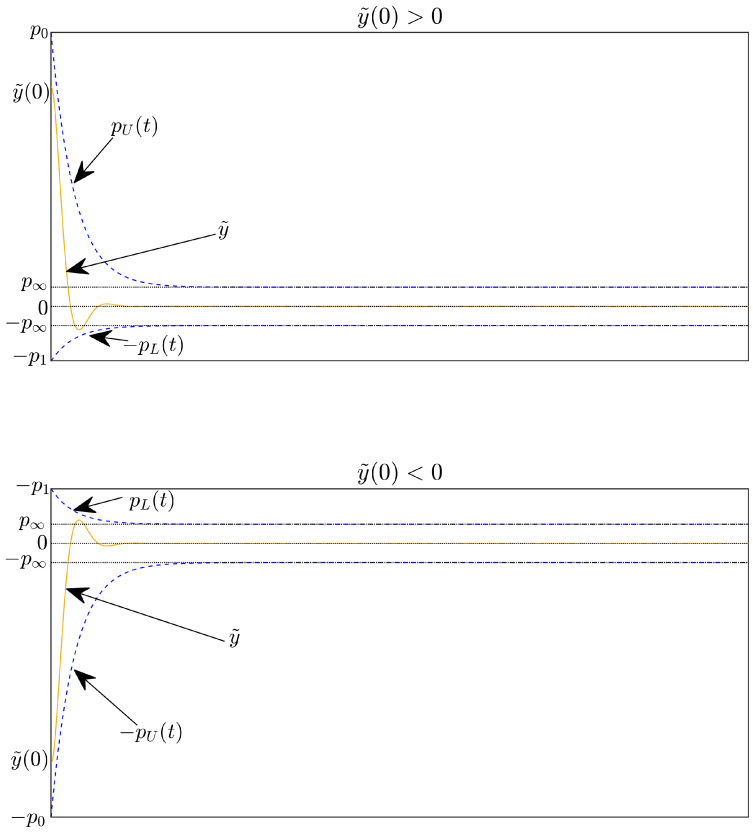
The prescribed tracking error behavior definition.

From the objective of the proposed controller, the prescribed performance indicates that the tracking error $\tilde{y}$ is confined within a preset region as follows:

(19) $$-p_{L}\left(t\right)<\tilde{y}\mathrm{sign}\left({\tilde{y}}_{0}\right)<p_{U}\left(t\right)$$

with

$$\begin{array}{c} \left\{\begin{array}{c} p_{U}\left(t\right)=\left(p_{0}-p_{\infty }\right)exp\left(-rt\right)+p_{\infty }\\ p_{L}\left(t\right)=\left(p_{1}-p_{\infty }\right)exp\left(-rt\right)+p_{\infty } \end{array}\right., \end{array}$$

where ${\tilde{y}}_{0}$ is the initial tracking error, $p_{U}\left(t\right)$ and $p_{L}\left(t\right)$ are the PPFs. The $p_{U}\left(t\right)$ and $p_{L}\left(t\right)$ are defined as [34]: $p_{U}\left(t\right)$ and $p_{L}\left(t\right):R_{+}\to R_{+}$ are smoothly, positive, and decreasing functions, respectively satisfying $\lim_{t\to \infty }p_{U}\left(t\right)=p_{\infty }>0$, $\lim_{t\to \infty }p_{L}\left(t\right)=p_{\infty }>0$, and $R_{+}$ is a set of positive real numbers. $p_{0}>\left|{\tilde{y}}_{0}\right|>0,p_{0}⩾p_{1}⩾p_{\infty }$, *r* are positive constants used to tune the specified performance region. Figure 2 illustrates the prescribed tracking error behavior definition.

**Remark** **2.** *It is noted that, when the sign of the initial error changes, the position of the lower and upper bounds will be reversed through the signum function. The PPFs $p_{U}\left(t\right)$ and $p_{L}\left(t\right)$ are assigned to the upper and lower bounds of the specified performance domain. $p_{U}\left(t\right)$ limits the convergence rate and the maximum allowable size of the tracking error $\tilde{y}$ at the steady state of the upper boundary. $p_{L}\left(t\right)$ limits the maximum allowable boundary of the overshoot and the maximum allowable size of the tracking error $\tilde{y}$ at the steady state of the lower boundary. The constant $p_{\infty }$ represents the maximum allowable size of the tracking error $\tilde{y}$ at the steady-state, the maximum overshoot of $\tilde{y}$ is prescribed as less than $p_{1}$, and the constraint on the convergence rate of $\tilde{y}$ depends on the decreasing rate of $p_{U}\left(t\right)$ which is adjusted by r. Hence, the appropriate selection of the PPFs $\left(p_{U}\left(t\right),\;p_{L}\left(t\right)\right)$, which imposes the output trajectory of the system.*

In order to assure that the prescribed performance is maintained, an ETF is used to convert the constrained error dynamics into the equivalent unconstrained dynamics. More specifically, we define:

(20) $$\tilde{y}=p\left(t\right)T\left(\tilde{z}\right)$$

where $\tilde{z}$ is a transformed error, and

$$\begin{array}{c} p\left(t\right)=\left\{\begin{array}{c} p_{U}\left(t\right)\;\;\;\mathrm{if}\;\mathrm{sign}\left(\tilde{y}.\tilde{y}\left(0\right)\right)>0\\ p_{L}\left(t\right)\;\;\;\mathrm{if}\;\mathrm{sign}\left(\tilde{y}.\tilde{y}\left(0\right)\right)<0 \end{array}\right., \end{array}$$

and $T(\tilde{z})$ is an ETF that possesses the following properties:

- $T(\tilde{z})$ is a smooth and strictly increasing function;
$-1<T(\tilde{z})<1$;
- $T(\tilde{z})=0$ if $\tilde{z}=0$;
- $\left\{\begin{array}{c} \lim_{\tilde{z}\to -\infty }T\left(\tilde{z}\right)=-1\\ \lim_{\tilde{z}\to +\infty }T\left(\tilde{z}\right)=1 \end{array}\right.$.

Owing to the definition of the PPF, when $\tilde{y}\left(0\right)>0$ and $\tilde{y}>0$, we have $0<T(\tilde{z})<1$ and $p_{U}\left(t\right)>0$. Hence,

(21) $$0<p_{U}\left(t\right)T\left(\tilde{z}\right)<p_{U}\left(t\right)$$

When $\tilde{y}\left(0\right)>0$ and $\tilde{y}<0$, we have $-1<T(\tilde{z})<0$ and $p_{L}\left(t\right)>0$. Hence,

(22) $$-p_{L}\left(t\right)<p_{L}\left(t\right)T\left(\tilde{z}\right)<0$$

From Equations (20)–(22) whenever $\tilde{y}\left(0\right)>0$, we can obtain

(23) $$-p_{L}\left(t\right)<\tilde{y}<p_{U}\left(t\right)$$

Otherwise, when $\tilde{y}\left(0\right)<0$ and $\tilde{y}<0$, we have

(24) $$-p_{U}\left(t\right)<p_{U}\left(t\right)T\left(\tilde{z}\right)<0$$

and, if $\tilde{y}\left(0\right)<0$ and $\tilde{y}>0$, when we have

(25) $$0<p_{L}\left(t\right)T\left(\tilde{z}\right)<p_{L}\left(t\right)$$

From Equations (20), (24) and (25) whenever $\tilde{y}\left(0\right)<0$, we can obtain

(26) $$-p_{U}\left(t\right)<\tilde{y}<p_{L}\left(t\right)$$

From Equations (23) and (26), we completely can achieve Equation (19), which represents a mathematical interpretation of the control objective to achieve the prescribed tracking error behavior over transient and steady-state scenarios.

The ETF is designed as

(27) $$T\left(\tilde{z}\right)=\frac{2}{\pi }\arctan\left(\tilde{z}\right)$$

Based on Equations (20) and (27), the transformed error $\tilde{z}$ can be calculated as follows:

(28) $$\tilde{z}=\tan\left(\frac{\pi \tilde{y}}{2p(t)}\right)$$

Taking time derivative of $\arctan(\tilde{z})$, we have

(29) $$\begin{array}{c} {\left(\arctan(\tilde{z})\right)}^{\prime }=\frac{\dot{\tilde{z}}}{1+{\tilde{z}}^{2}} \end{array}$$

where $\dot{\tilde{z}}$ is the time derivative of $\tilde{z}$.

Taking the time derivative of Equation (20) and using Equations (27) and (29), we have

(30) $$\begin{array}{c} \dot{\tilde{y}}=\dot{p}\left(t\right)T\left(\tilde{z}\right)+p\left(t\right)\dot{T}\left(\tilde{z}\right)\\ \;\;\;=\dot{p}\left(t\right)\frac{2}{\pi }\arctan\left(\tilde{z}\right)+p\left(t\right)\frac{2}{\pi }\frac{\dot{\tilde{z}}}{1+{\tilde{z}}^{2}} \end{array}$$

where $\dot{p}\left(t\right)=\left\{\begin{array}{c} {\dot{p}}_{U}\left(t\right)\;\;\mathrm{if}\;\mathrm{sign}\left(\tilde{y}.\tilde{y}\left(0\right)\right)>0\\ {\dot{p}}_{L}\left(t\right)\;\;\;\mathrm{if}\;\mathrm{sign}\left(\tilde{y}.\tilde{y}\left(0\right)\right)<0 \end{array}\right.$.

From Equation (30), the time derivative of transformed error $\tilde{z}$ is obtained:

(31) $$\dot{\tilde{z}}=\frac{\pi \left(1+{\tilde{z}}^{2}\right)}{2p(t)}\left(\dot{\tilde{y}}-\frac{2\dot{p}\left(t\right)}{\pi }\arctan\left(\tilde{z}\right)\right)$$

Taking the second-order time derivative of $\arctan(\tilde{z})$, we have

(32) $${\left(\arctan(\tilde{z})\right)}^{\prime \prime }=\frac{\ddot{\tilde{z}}\left(1+{\tilde{z}}^{2}\right)-2\tilde{z}{\dot{\tilde{z}}}^{2}}{{\left(1+{\tilde{z}}^{2}\right)}^{2}}$$

where $\ddot{\tilde{z}}$ is the second-order time derivative of $\tilde{z}$.

Taking the second-order time derivative of Equation (20) and using Equations (27), (29) and (32), we have

(33) $$\begin{array}{c} \ddot{\tilde{y}}={\left(\dot{p}\left(t\right)T\left(\tilde{z}\right)+p\left(t\right)\dot{T}\left(\tilde{z}\right)\right)}^{\prime }\;\\ \;\;\;=\ddot{p}\left(t\right)T\left(\tilde{z}\right)+2\dot{p}\left(t\right)\dot{T}\left(\tilde{z}\right)+p\left(t\right)\ddot{T}\left(\tilde{z}\right)\\ \;\;=\frac{2}{\pi }\left(\ddot{p}\left(t\right)\arctan\left(\tilde{z}\right)+\frac{2\dot{p}\left(t\right)\dot{\tilde{z}}}{1+{\tilde{z}}^{2}}-\frac{2p\left(t\right)\tilde{z}{\dot{\tilde{z}}}^{2}}{{\left(1+{\tilde{z}}^{2}\right)}^{2}}\right)\\ \;\;\;\;\;\;\;\;+\frac{2p(t)}{\pi }\frac{\ddot{\tilde{z}}}{\left(1+{\tilde{z}}^{2}\right)} \end{array}$$

where $\ddot{p}\left(t\right)=\left\{\begin{array}{c} {\ddot{p}}_{U}\left(t\right)\;\;if\;\mathrm{sign}\left(\tilde{y}.\tilde{y}\left(0\right)\right)>0\\ {\ddot{p}}_{L}\left(t\right)\;\;\;if\;\mathrm{sign}\left(\tilde{y}.\tilde{y}\left(0\right)\right)<0 \end{array}\right.$.

From Equation (33), we can derive the second-order time derivative of transformed error as follows:

(34) $$\ddot{\tilde{z}}=A\left(\ddot{\tilde{y}}-N\right)$$

where $A=\frac{\pi \left(1+{\tilde{z}}^{2}\right)}{2p(t)}>0$, $N=\frac{2}{\pi }\left(\ddot{p}\left(t\right)\arctan\left(\tilde{z}\right)+\frac{2\dot{p}\left(t\right)\dot{\tilde{z}}}{1+{\tilde{z}}^{2}}-\frac{2p\left(t\right)\tilde{z}{\dot{\tilde{z}}}^{2}}{{\left(1+{\tilde{z}}^{2}\right)}^{2}}\right)$.

### 3.5. Proposed Controller Design

For system (5), the sliding manifold is constructed by using the transformed position error and the transformed velocity error as follows:

(35) $$s=\dot{\tilde{z}}+\lambda^{*}sig{\left(\tilde{z}\right)}^{\alpha }+\omega^{*}sig{\left(\tilde{z}\right)}^{\beta }$$

where $\lambda^{*}=\frac{2\lambda }{1+e^{-\eta \left(\left|\tilde{z}\right|-\gamma \right)}}$, $\omega^{*}=\frac{2\omega }{1+e^{\vartheta \left(\left|\tilde{z}\right|-\gamma \right)}}$, λ>0,η>0,ω>0,σ>0, α>1, 0<β<1, $\gamma ={\left(\omega /\lambda \right)}^{1/\left(\alpha -\beta \right)}$.

The time derivative of the selected sliding mode surface is:

(36) $$\begin{array}{c} \dot{s}=\ddot{\tilde{z}}+\frac{2\lambda_{i}\alpha }{1+e^{-\eta \left(\left|\tilde{z}\right|-\gamma \right)}}{\left|\tilde{z}\right|}^{\alpha -1}\dot{\tilde{z}}+\frac{2\lambda \eta e^{-\eta \left(\left|\tilde{z}\right|-\gamma \right)}}{{\left(1+e^{-\eta \left(\left|\tilde{z}\right|-\gamma \right)}\right)}^{2}}{\left|\tilde{z}\right|}^{\alpha }\dot{\tilde{z}}\\ +\frac{2\omega \beta }{1+e^{\vartheta \left(\left|\tilde{z}\right|-\gamma \right)}}{\left|\tilde{z}\right|}^{\beta -1}\dot{\tilde{z}}-\frac{2\omega \vartheta e^{\vartheta \left(\left|\tilde{z}\right|-\gamma \right)}}{{\left(1+e^{\vartheta \left(\left|\tilde{z}\right|-\gamma \right)}\right)}^{2}}{\left|\tilde{z}\right|}^{\beta }\dot{\tilde{z}}, \end{array}$$

Equation (36) is rewritten as:

(37) $$\dot{s}=\ddot{\tilde{z}}+M_{\tilde{z}}$$

where $M_{\tilde{z}}=\frac{2\lambda_{i}\alpha }{1+e^{-\eta \left(\left|\tilde{z}\right|-\gamma \right)}}{\left|\tilde{z}\right|}^{\alpha -1}\dot{\tilde{z}}+\frac{2\lambda \eta e^{-\eta \left(\left|\tilde{z}\right|-\gamma \right)}}{{\left(1+e^{-\eta \left(\left|\tilde{z}\right|-\gamma \right)}\right)}^{2}}{\left|\tilde{z}\right|}^{\alpha }\dot{\tilde{z}}+\frac{2\omega \beta }{1+e^{\vartheta \left(\left|\tilde{z}\right|-\gamma \right)}}{\left|\tilde{z}\right|}^{\beta -1}\dot{\tilde{z}}-\frac{2\omega \vartheta e^{\vartheta \left(\left|\tilde{z}\right|-\gamma \right)}}{{\left(1+e^{\vartheta \left(\left|\tilde{z}\right|-\gamma \right)}\right)}^{2}}{\left|\tilde{z}\right|}^{\beta }\dot{\tilde{z}}$.

Substituting Equations (5) and (34) into Equation (37) yields:

(38) $$\dot{s}=A\left(g-\frac{\hat{\mu }}{y_{1}^{2}}U^{2}+\Delta \left(y,\delta ,t\right)-{\ddot{y}}_{r}-N\right)+M_{\tilde{z}}$$

The proposed controller is designed to obtain the control object as follows:

(39) $$U=\sqrt{\frac{y_{1}^{2}}{\hat{\mu }}\left(u_{eq}+u_{ob}+u_{r}\right)}.$$

where the elements of *U* are designed as:

$$\begin{array}{c} \left\{\begin{array}{c} u_{eq}=g-{\ddot{y}}_{r}-N+A^{-1}M_{\tilde{z}}\\ u_{ob}=\hat{\Delta }\\ u_{r}=\sigma_{2}s+\left(\bar{\delta }+\kappa_{2}\right)\mathrm{sign}\left(s\right). \end{array}\right. \end{array}$$

where $\bar{\delta }>0$ is the bounded value of the estimation error of the disturbance observer $\left(\bar{\delta }⩾\left|\Delta \left(y,\delta ,t\right)-\hat{\Delta }\right|\right)$, and $\kappa_{2}$ and $\sigma_{2}$ are positive constants.

**Figure 3:**
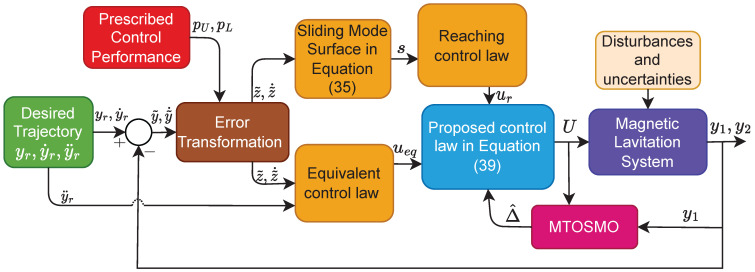
The block diagram of the proposed control system.

The block diagram of the proposed control system is shown in Figure 3.

The following theorem summarizes the control design procedure.

**Theorem** **1.** *For the magnetic levitation system presented in Equation (5), if the control input signal is designed as (39), then the system (5) is finite-time stable. Furthermore, the maximum overshoot and steady state of position tracking error are guaranteed in prescribed control performance.*

**Proof** **of** **Theorem** **1.** Applying the control signal system (39) to (38) gains:

(40) $$\dot{s}=A\left(\Delta \left(y,\delta ,t\right)-\hat{\Delta }-\left(\bar{\delta }+\kappa_{2}\right)\mathrm{sign}\left(s\right)-\sigma_{2}s\right)$$

Considering the following Lyapunov function $V_{2}=\frac{p_{0}}{\pi }s^{2}$, then we see that:

(41) $${\dot{V}}_{2}=\frac{2p_{0}}{\pi }s\dot{s}$$

By substituting Equation (40) into Equation (41), we obtain:

(42) $$\begin{array}{c} {\dot{V}}_{2}=\frac{2p_{0}}{\pi }sA\left(\begin{array}{c} \Delta \left(y,\delta ,t\right)-\hat{\Delta }\\ -\left(\bar{\delta }+\kappa_{2}\right)\mathrm{sign}\left(s\right)-\sigma_{2}s \end{array}\right)\\ \;\;\;\;\;⩽\frac{2p_{0}}{\pi }sA\left(\bar{\delta }-\left(\bar{\delta }+\kappa_{2}\right)\mathrm{sign}\left(s\right)-\sigma_{2}s\right)\\ \;\;\;\;\;⩽-\frac{2p_{0}}{\pi }sA_{\min}\left(\kappa_{2}\mathrm{sign}\left(s\right)+\sigma_{2}s\right)\\ \;\;\;\;\;⩽-\kappa_{2}\left|s\right|-\sigma_{2}s^{2}\\ \;\;\;\;\;⩽-\kappa_{2}\sqrt{\frac{\pi }{p_{0}}}{\left|V_{2}\right|}^{1/2}-\frac{\pi \sigma_{2}}{p_{0}}V_{2}⩽0 \end{array}$$

From Equations (41) and (42), we can conclude that system (5) is finite-time stable according to [43]. Thus, the sliding variables $s,\dot{s}$ can reach the sliding manifold in finite-time (s→0 and $\dot{s}\to 0$) that means $\tilde{z},\dot{\tilde{z}}$ converge to its equilibrium point in finite time. Consequently, the position tracking error $\tilde{y}$ is converged to the equilibrium point in finite time and guaranteed in prescribed control performance. The proof is completed. □

## 4. Simulation and Experimental Results

In this section, two examples include a simulation and an experiment on the laboratory magnetic levitation model to demonstrate the improved performance of the developed control system.

### 4.1. Simulation Results

The MLS is established according to [4]. The considered system parameters are stated in Table 1. The maximum control voltage is $U_{\max}<5\;V$, and the sampling time is $1.10^{-3}$ s.

A magnetic ball started in a certain position and followed a prescribed trajectory at the start of the simulation. The desired trajectory as sinusoidal is planned below:

(43) $$\begin{array}{c} y_{r}=15+3\sin\left(0.2\pi t\right)\;\;\;\;\;\left(\mathrm{mm}\right) \end{array}$$

With the assumed disturbance δ(t)=2sin(t), the upper bound of uncertain terms is defined by:

(44) $$\begin{array}{c} \left|\Delta \left(y,\delta ,t\right)\right|\leq \frac{\left|\mu -\hat{\mu }\right|}{y_{\min}^{2}}U_{\max}^{2}+\delta_{max}\left(t\right)=2.7 \end{array}$$

where $y_{r\min}=12\left(\mathrm{mm}\right)$, and the initial value of $y_{0}=26\left(\mathrm{mm}\right)$.

In addition, to check the influence of sensor measurement noise, an external noise in the form of Gaussian random noise with the variance of 0.0001 is added to the velocity sensor. Thus, the simulation case will be similar to the real system where there is always noise from the measuring device.

The root-mean-square tracking error (RMSTE) is calculated as follows:

(45) $$\begin{array}{c} \mathrm{RMSTE}=\sqrt{\frac{1}{N}\sum_{i=1}^{N}{\left(y_{ri}-y_{i}\right)}^{2}} \end{array}$$

in which *N* is the number of samples to be taken into account in this calculation. $y_{ri}$ and $y_{i}$ are respectively the desired trajectory and the real trajectory at the time index *i*th.

To facilitate the evaluation of the accuracy of the controllers, the RMSTE is calculated according to Equation (45), in the time interval after convergence (3rd to 30th second).

In the simulation example, the control parameters of the four controllers were selected as in Table 2.

We compared the simulated control performance of the four controllers, PID, SMC, GFTSMC (11), and the proposed controller, to determine which is the most effective.

The PID controller has control voltage as follows:

(46) $$\begin{array}{c} U=K_{p}\tilde{y}+K_{i}\int \tilde{y}+K_{d}\dot{\tilde{y}} \end{array}$$

where $K_{p},\;K_{i},\;K_{d}$ are control gains.

The SMC controller has control voltage as follows:

(47) $$\begin{array}{c} U=\sqrt{\frac{y_{1}^{2}}{\hat{\mu }}\left(g+M_{\tilde{y}}-{\ddot{y}}_{r}+\sigma s+\left(\bar{\Delta }+\kappa \right)sign\left(s\right)\right)} \end{array}$$

where $s=\dot{\tilde{y}}+c\tilde{y}$ is a linear sliding surface, c>0. κ and σ are positive constants.

The simulation process and the result evaluation are carried out in four steps:

- Step 1: simulates and evaluates the approximation ability of the proposed observer through a comparison between its approximation ability and the conventional TOSMO;
- Step 2: investigates the management of the terms of the proposed PPC including maximum overshoot and steady-state of the controlled errors;
- Step 3: compares the tracking accuracy, maximum overshoot and steady-state of the controlled errors among the four control methods through figures plotted from MATLAB and RMS methods;
- Step 4: considers the chattering behavior that appeared in the control signal of the four methods.

**Figure 4:**
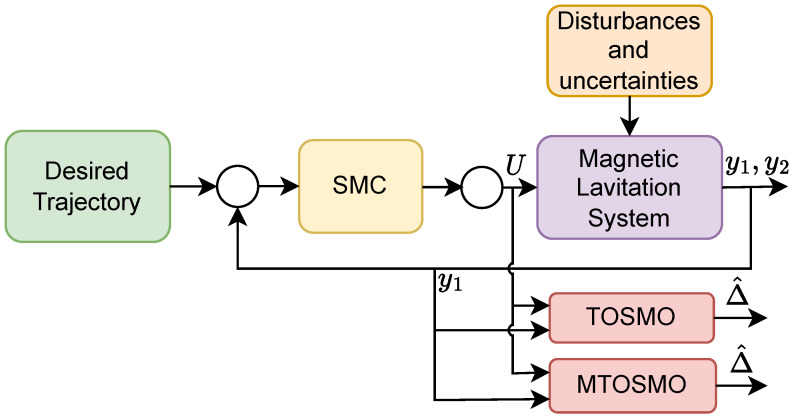
The setting for comparing two observers.

To compare the estimation performance of the TOSMO and MTOSMO, we use the SMC (47) to control the MLS, while the two observers are run in parallel with SMC to compare their outputs as shown in Figure 4. The TOSMO is designed as [17]:

(48) $$\begin{array}{c} \left\{\begin{array}{c} {\dot{\hat{y}}}_{1}=\pi_{1}{\left[{\bar{y}}_{1}\right]}^{{}^{\frac{2}{3}}}+{\hat{y}}_{2}\\ {\dot{\hat{y}}}_{2}=g-\frac{\hat{\mu }}{y_{1}^{2}}U^{2}+\pi_{2}{\left[{\bar{y}}_{1}\right]}^{{}^{\frac{1}{3}}}+\hat{\Delta }\\ \dot{\hat{\Delta }}=\pi_{3}sign\left({\bar{y}}_{1}\right) \end{array}\right. \end{array}$$

where ${\hat{y}}_{1}$, ${\hat{y}}_{2}$, and $\hat{\Delta }$ are the estimated value of $y_{1}$, $y_{2}$, $\Delta \left(y,\delta ,t\right)$, respectively. $\pi_{i}\left(i=1,2,3\right)$ represents observer gains. The parameters of SMC and MTOSMO are selected in Table 2, and the parameters of TOSMO are set the same as MTOSMO.

**Figure 5:**
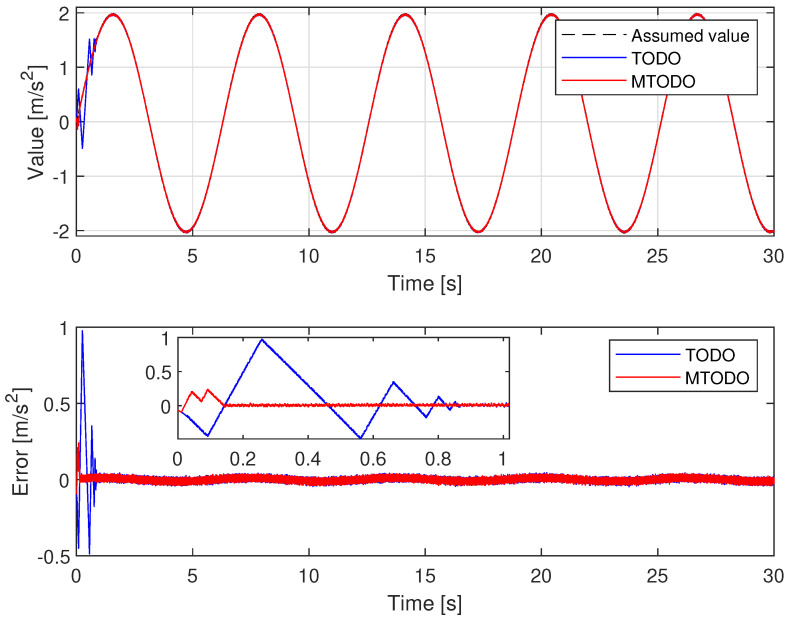
Time evolution of observer output.

From Figure 5, the uncertain components can be approximated accurately by both observers. However, the proposed TOSMO has a faster convergence speed than the traditional TOSMO. This is absolutely necessary to avoid causing a delay to the control system. Therefore, it plays a role in improving control performance.

**Figure 6:**
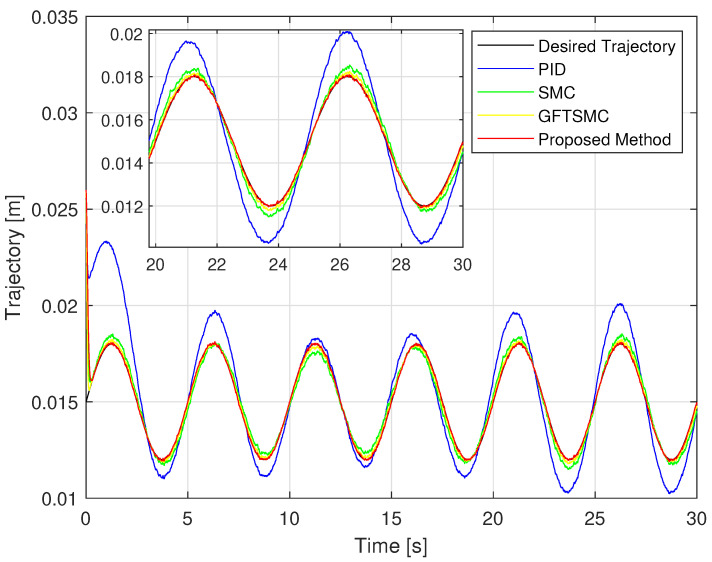
The controlled ball trajectory under four controllers: planned trajectory and actual trajectory.

**Figure 7:**
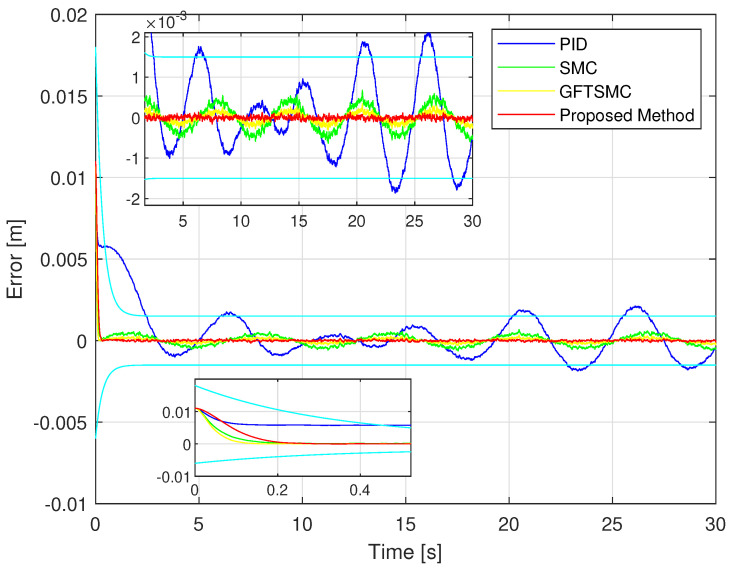
Time evolution of the trajectory errors of the controlled ball using four control methods.

Looking at Figure 6 and Figure 7, it is clear that the proposed controller has the smallest maximum overshoot and the smallest controlled errors at a steady state. Both of these metrics can be predefined by adjusting the design parameters of the PPFs in Equation (19). In contrast, we cannot manage the maximum overshoot as well as the tracking error at the steady state of the controlled magnetic ball through the remaining three controllers. Specifically, the PID controller can not satisfy both stated terms. In addition, comparing the boundary values of the proposed PPF with the boundary values of the existing PPFs [36,38,39,40], we found that the boundary values of conventional PPFs are designed largely up to $10^{-2}$. It is noted that the existing controllers can almost obtain this accuracy level. Therefore, it does not see clearly the management of the prescribed performance of the PPCs, while the boundary values of the proposed PPF are designed smaller than that of conventional PPFs, and its value is $10^{-3}$.

The simulation tracking performance of the controlled ball from four different control methods is illustrated in Figure 6 and Figure 7. It can be seen clearly in Figure 6 that the trajectory deviation of the magnetic ball controlled by the PID versus the desired trajectory is the largest, and the GFTSMC provides a smaller trajectory deviation of the ball than that of the PID and the SMC, while the proposed controller provides the smallest trajectory deviation of the ball among the four methods. We investigate in detail the tracking accuracy illustrated in Figure 7 and Table 3 and recognize that the proposed controller controls the ball with the smallest steady-state errors, $\mathrm{RMSTE}=4.2119\times 10^{-5}$, and the smallest maximum overshoot that satisfied the predefined prescribed performance; the SMC and the GFTSMC control the ball with seemingly equivalent performance, the control errors of these two methods are $\mathrm{RMSTE}=3.1097\times 10^{-4}$ and $\mathrm{RMSTE}=1.979\times 10^{-4}$, respectively; the PID controls the ball with the largest control error, $\mathrm{RMSTE}=1.0034\times 10^{-3}$ and the largest maximum overshoot that do not satisfy the predefined operation domain.

**Figure 8:**
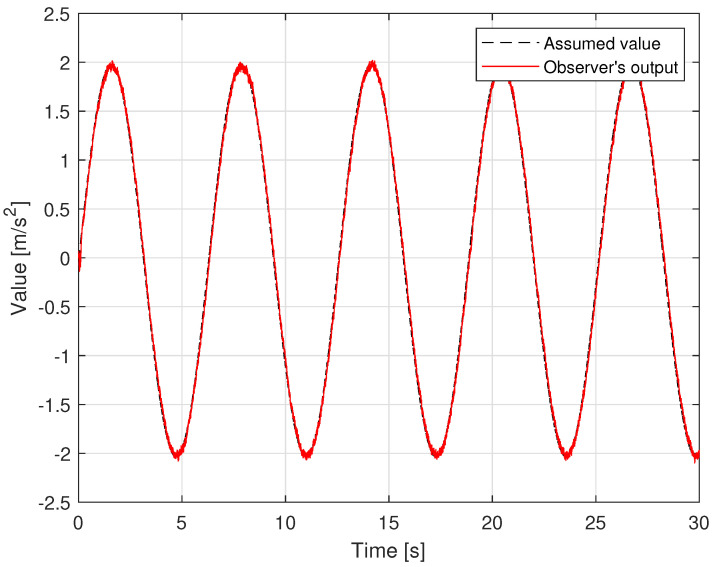
Time evolution of observer output versus assumed disturbance.

Regarding the problem of oscillations in the control voltage, all four control methods appear oscillation phenomena. Due to the influence of sensor measurement noise, even a linear controller like a PID also has oscillations. The fluctuation amplitude of the control voltage of the SMC and the GFTSMC is almost equivalent because both methods apply the same sliding value, which is chosen to be greater than or equal to the upper bound of the uncertain components, $\bar{\Delta }+\kappa =3.8$, to compensate for the effects of the uncertain elements. As shown in Figure 8, the suggested TOSMO can estimate accurately and quickly the assumed uncertain terms.

**Figure 9:**
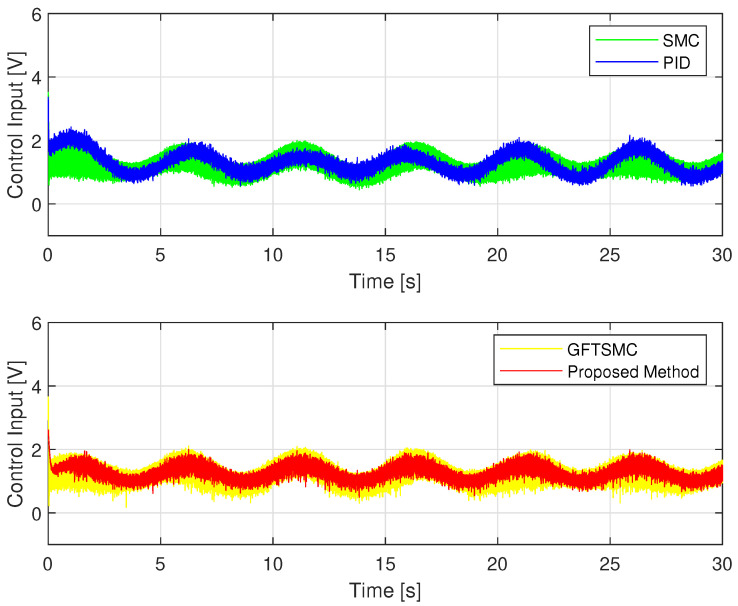
Time evolution of control voltage from four control methods.

Thanks to the TOSMO’s accurate information, the developed controller applied a smaller sliding value than that of the SMC and the GFTSMC. Therefore, the fluctuation amplitude of its control voltage is smaller than that of the two remaining methods as shown in Figure 9.

Simulation results show that the developed controller has the best control performance among the compared control algorithms.

### 4.2. Experimental Results

In order to test the effectiveness of the proposed control algorithm in the practical control system, an experiment on MLS was implemented.

**Figure 10:**
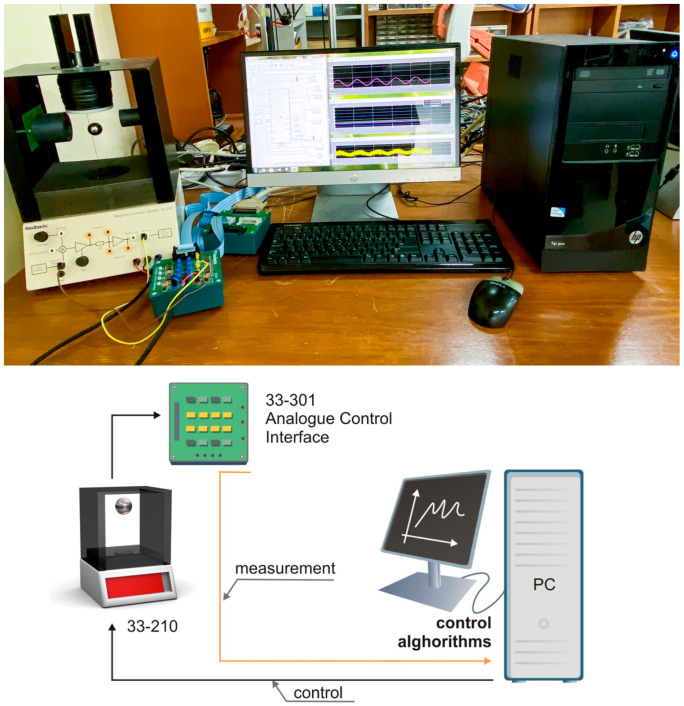
Configuration of experimental system.

The experimental MLS was produced by Feedback Instrument, as configured in Figure 10. The experimental system includes a mechanical unit (model Feedback 33-210) and an analog control interface (Feedback model 33-301). A PCI1711 I/O card was inserted into a PCI computer slot, and then it was connected to the feedback SCSI adapter box by the SCSI cable. The control programming was implemented by using MATLAB/Simulink, Real-Time Workshop (RTW), Microsoft Visual C++ Professional, Control Toolbox, and Real-Time Windows Target.

For more information on installing this experimental system, readers can refer to it in [4].

The maximum control voltage is $U_{\max}<5\;V$, and the sampling time is $1.10^{-3}$ s.

The magnetic ball started in a specific position and followed a prescribed trajectory when the experiment began. There were two desired trajectories: a sinusoidal as Equation (43) and a rest-to-rest line with an upper value of 17mm and lower value 12mm. There was an assumption that the disturbance δ(t)=2sin(t) would affect the system.

The control parameters of the four separate controllers selected in Table 2 are also used for the experimental example.

**Figure 11:**
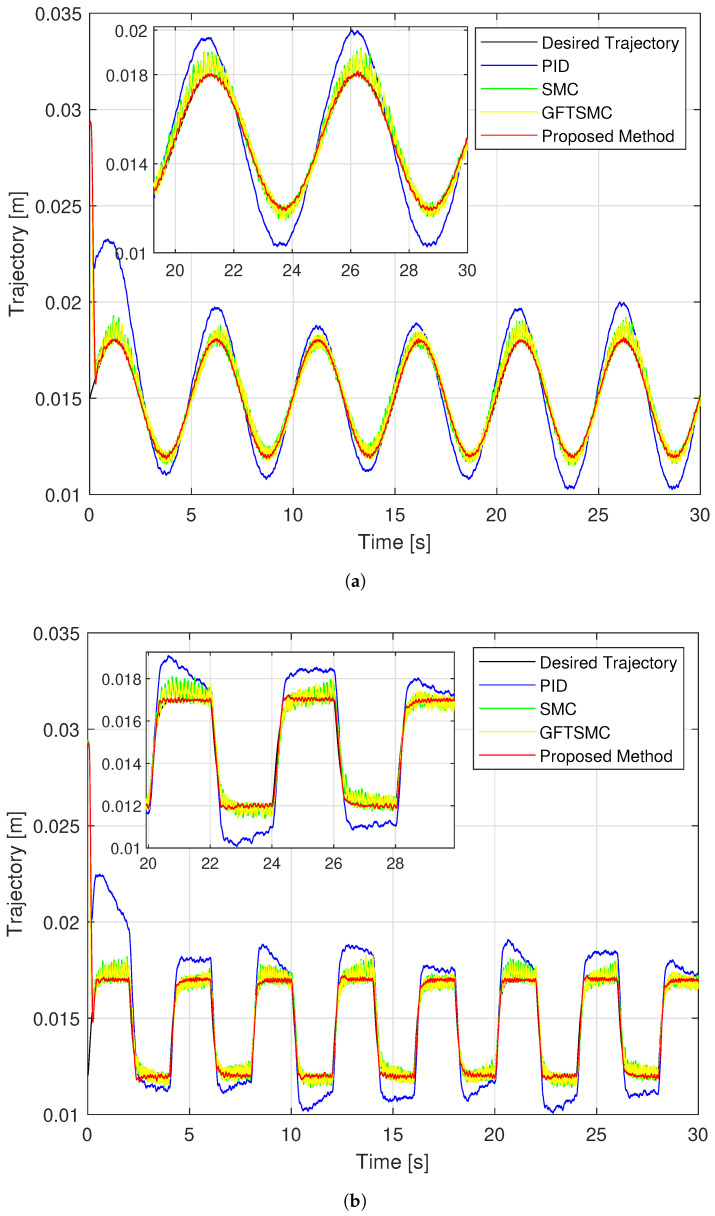
Comparison of the controlled ball trajectory with the desired trajectory using four separate controllers; (**a**) in case of sinusoidal orbit; (**b**) in case of a rest-to-rest line.

**Figure 12:**
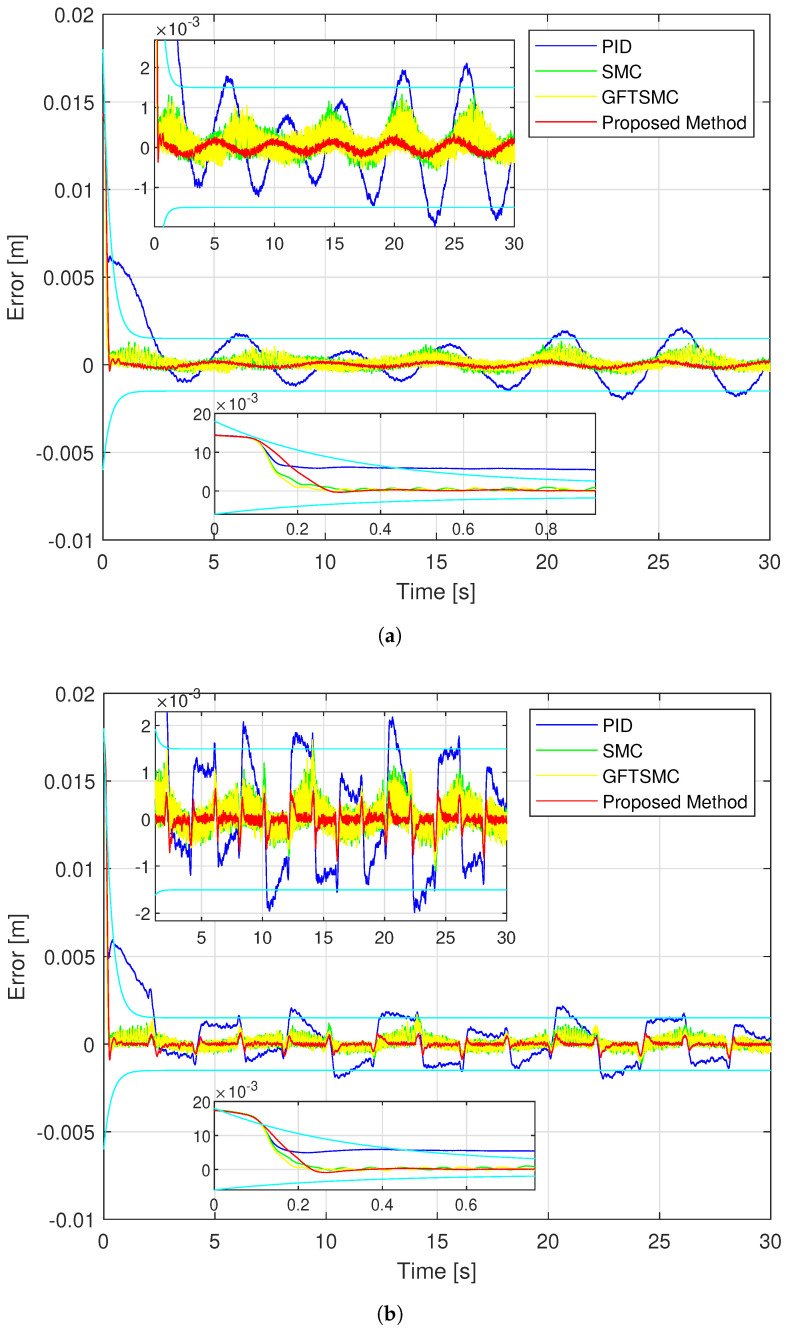
The trajectory errors of the controlled ball under four controllers; (**a**) in case of sinusoidal orbit; (**b**) in case of a rest-to-rest line.

**Figure 13:**
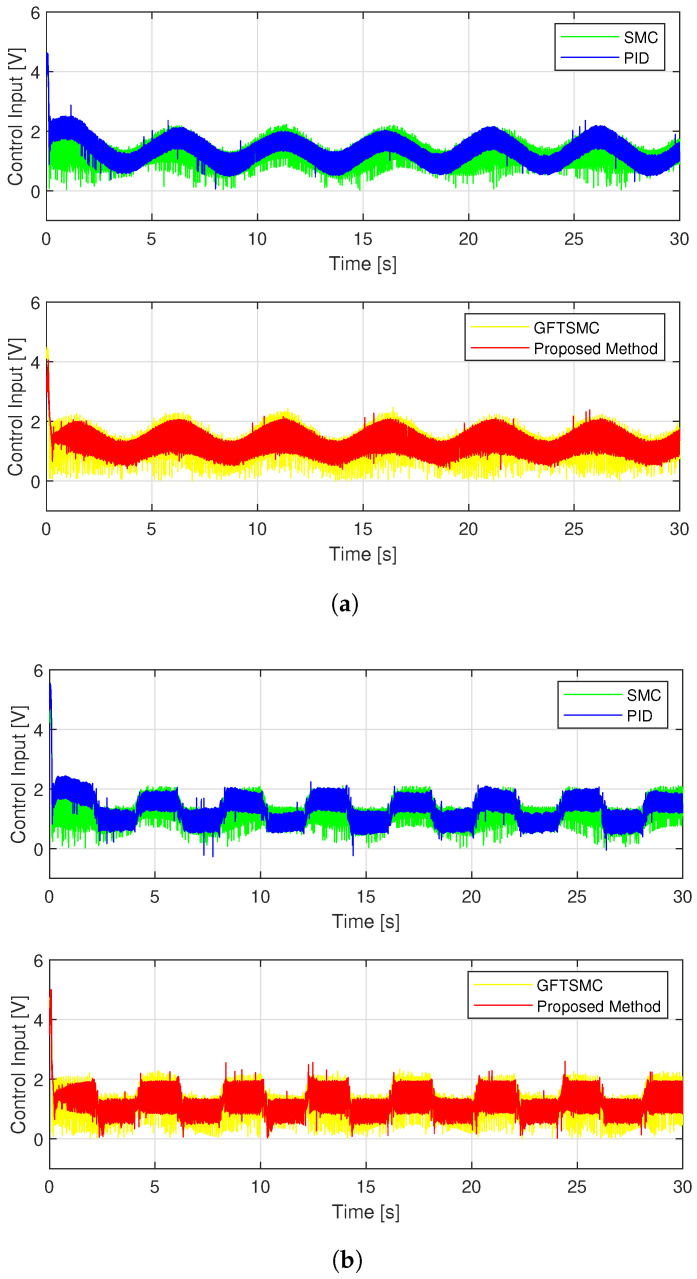
Control voltage of four controllers; (**a**) in case of sinusoidal orbit; (**b**) in case of a rest-to-rest line.

The experimental performance provided by four control methods for a magnetic ball by tracking a sinusoidal and a rest-to-rest line is exhibited in Figure 11, Figure 12 and Figure 13. The experiment process and results evaluation are carried out in three steps from step 2 to step 4 as in the simulation example.

Figure 11 and Figure 12 show that the proposed controller has the smallest maximum overshoot and the smallest controlled errors at a steady state, similar to the simulation results. Neither of these conditions can be satisfied by PID, hence the ball controlled by the PID can not track the reference trajectory well. The SMC and the GFTSMC cannot also obtain the controlled errors with small oscillations. It is seen that, at the 14th second, both SMC and GFTSMC do not satisfy a prescribed performance. Consequently, the ball controlled by the SMC and the GFTSMC will be vibrated around its operation point.

As shown in Figure 11, the proposed controller provides the smallest trajectory disparity of the magnetic ball among the four methods, the GFTSMC provides a smaller trajectory disparity of the ball than the PID or the SMC, and the PID provides the largest trajectory disparity of the ball. From Figure 12 and Table 4, the proposed controller also achieves the smallest steady-state errors with two different types of orbits. Apparently, it provided a control efficiency that was within a pre-specified boundary. The SMC and the GFTSMC achieve control efficiency with equivalent accuracy. With two different types of orbits, the maximum overshoot of the PID does not satisfy the predefined operation domain. The PID achieves the worst accuracy with both trajectories.

**Figure 14:**
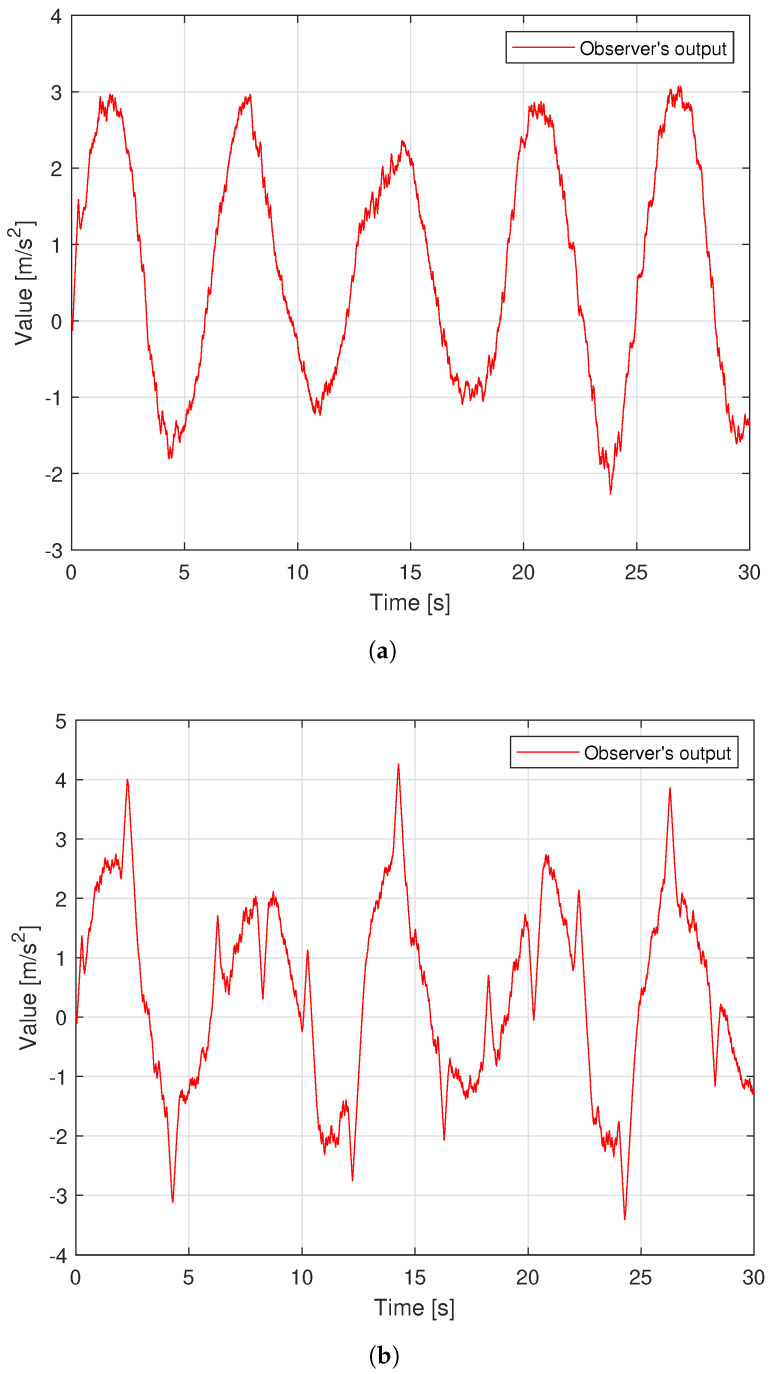
Time evolution of the MTOSMO’s output; (**a**) in case of sinusoidal orbit; (**b**) in case of a rest-to-rest line.

The noise in sensor measurement appears to be influencing all four control methods, which cause oscillations in control voltage. With two different types of orbits, the fluctuation amplitude of the control voltage of each controller is similar to the simulation results, as shown in Figure 13. Figure 14 shows the time evolution of the proposed TOSMO’s output.

In a comparison of the experimental performance, the developed controller also stands out as the best controller.

**Remark** **3.** *From the simulation and experimental results, we can see that the tracking performance of the proposed controller is guaranteed within a prescribed performance in both cases. In the experiment, the tracking accuracy of the proposed controller has slightly reduced compared to the simulation case; however, it is still at a high tracking accuracy. The chattering behavior in the control signal of the proposed controller in the experimental case is not significantly increased compared to the simulation case. It is concluded that the proposed control method is effective to control MLSs.*

## 5. Some Remarkable Conclusions

We implemented a real-time PPC for MLSs subject to dynamical uncertainty and exterior perturbations. A modified function of GFTSMM based on the transformed errors of the proposed PPC was developed; hence, the tracking error variables quickly converge to the equilibrium point with the prescribed performance. Maximum overshoot and steady-state of the controlled errors have been prescribed in a predefined boundary. By using the designed observer, it is possible to know the approximate value of the entire uncertainty, which contributes to reducing chattering and improving control performance. The combination of the GFTSMC, the PPC, and the MTOSMO generated a novel PPC strategy ensuring a finite-time stable position of the controlled ball and the possibility of performing different orbit tracking missions with an impressive performance in the terms of tracking accuracy, fast convergence, stabilization, and chattering reduction in real time. With a simple design, the proposed strategy is suitable for real-time applications of MLSs. Mathematical proof using Lyapunov theory, a simulation, and an experimental example on a laboratory magnetic levitation model have both been used to demonstrate the proposed controller’s stability and effectiveness.

## Figures and Tables

**Table 1:** System parameters.

System Parameters	Value	Unit
*g*	9.81	m/s${}^{2}$
*m*	0.02	kg
λ	$2.48315625\times 10^{-5}$	Nm${}^{2}$/A${}^{2}$
*K*	1.05	A/V
$\hat{\mu }$	0.00136487	(N.m${}^{2}$/kg.V${}^{2}$)

**Table 2:** The control parameters.

Controller	Symbol	Value
PID	$K_{p},\;K_{i},\;K_{d}$	300,100,10
SMC	$c,\;\sigma ,\bar{\Delta }+\kappa$	20,50,3.8
GFTSMC	λ,ω,η,ϑ,α,β	10,10,1.1,2.2,1.1,0.8
	$\sigma_{1},\bar{\Delta }+\kappa_{1}$	20,50,3.8
Proposed Method	$p_{0},p_{1},p_{\infty },r$	0.023,0.006,0.0015,3
	λ,ω,η,ϑ,α,β	10,10,1.1,2.2,1.1,0.8
	$\sigma_{2},\bar{\delta }+\kappa_{2}$	0.13,0.1
	$\pi_{1},\pi_{2},\pi_{3},\rho$	5.45,3.67,6.6,100

**Table 3:** RMSTE using the four separate controllers.

Controller	RMSTE
PID	$1.0034\times 10^{-3}$
SMC	$3.1097\times 10^{-4}$
GFTSMC	$1.979\times 10^{-4}$
Proposed Method	$4.2119\times 10^{-5}$

**Table 4:** RMSTE of four controllers.

Controller	RMSTE in Case of Sinusoidal Orbit	RMSTE in Case of a Rest-to-Rest Line
PID	$1.0411\times 10^{-3}$	$1.1342\times 10^{-3}$
SMC	$3.6039\times 10^{-4}$	$3.7681\times 10^{-4}$
GFTSMC	$3.3052\times 10^{-4}$	$3.4805\times 10^{-4}$
Proposed Method	$1.2203\times 10^{-4}$	$1.9601\times 10^{-4}$

## Data Availability

The data sets generated and/or analyzed during the current study are available from the corresponding author on reasonable request.
